# CT-guided bone biopsy using electron density maps from dual-energy CT

**DOI:** 10.1016/j.radcr.2021.06.009

**Published:** 2021-07-01

**Authors:** Shota Yamamoto, Shunsuke Kamei, Kosuke Tomita, Chikara Fujita, Kazuyuki Endo, Shinichiro Hiraiwa, Terumitsu Hasebe

**Affiliations:** 1 Department of Radiology, Tokai University Hachioji Hospital, Tokai University School of Medicine, Tokyo, Japan; 2 Department of Radiological Technology, Tokai University Hachioji Hospital, Tokai University School of Medicine, Tokyo, Japan; 3 Department of Pathology, Tokai University Hachioji Hospital, Tokai University School of Medicine, Tokyo, Japan

**Keywords:** Bone metastasis, Computed tomography-guided intervention, Bone biopsy, Dual energy computed tomography, Electron density

## Abstract

Computed tomography (CT) -guided bone biopsy is a diagnostic procedure performed on the musculoskeletal system with a high diagnostic yield and low complications. However, CT-guided bone biopsy has the disadvantage that it is difficult to confirm the presence of tumor cells during the biopsy procedure. Recently, the clinical benefits of dual-energy CT (DECT) over single-energy CT have been revealed. DECT can provide material decomposition images including calcium suppression images, and effective atomic number (Z_eff_) and electron density (ED) maps. ED maps have been reported to indicate cellularity. A 61-year-old woman with a history of breast cancer surgery was admitted to our hospital and underwent a CT-guided bone biopsy of the right ilium using ED maps. As a result, she was diagnosed with breast cancer metastases of intertrabecular bone. A comparison of ED maps with a pathological specimen revealed that high ED values occurred exclusively in the tumor area with high cellularity. This study indicates that ED maps produced using DECT may have potential utility in the accurate identification of metastases with high cellularity in bone lesions.

## Introduction

Computed tomography (CT)-guided bone biopsy is a diagnostic procedure for obtaining a tissue sample with a high diagnostic yield and low complications [1]. However, CT-guided bone biopsy has the disadvantage that it is difficult to confirm the presence of viable tumor cells during the biopsy procedure. Therefore, there are some cases in which the procedure succeeded but the diagnosis remained unconfirmed or was incorrect [2,3].

Recently, the clinical benefits of dual-energy CT (DECT) over single-energy CT have been revealed [4]. DECT uses two separate X-ray photon energy spectra to integrate the assessment of materials with different attenuation properties at different energies. Dual-energy data (attenuation values at two energy spectra) can be used to reconstruct numerous types of material decomposition images including calcium suppression images, effective atomic number (Z_eff_) maps, and electron density (ED) maps [5]. It has been reported that ED maps may be used to describe different types of atoms, chemical bonds, and tissue compositions [6,7], which suggests that they may indicate cellularity [8]. Here, we report a case involving breast cancer bone metastasis, which was identified in a patient who underwent a CT-guided bone biopsy using ED maps.

## Case report

**Fig. 1:**
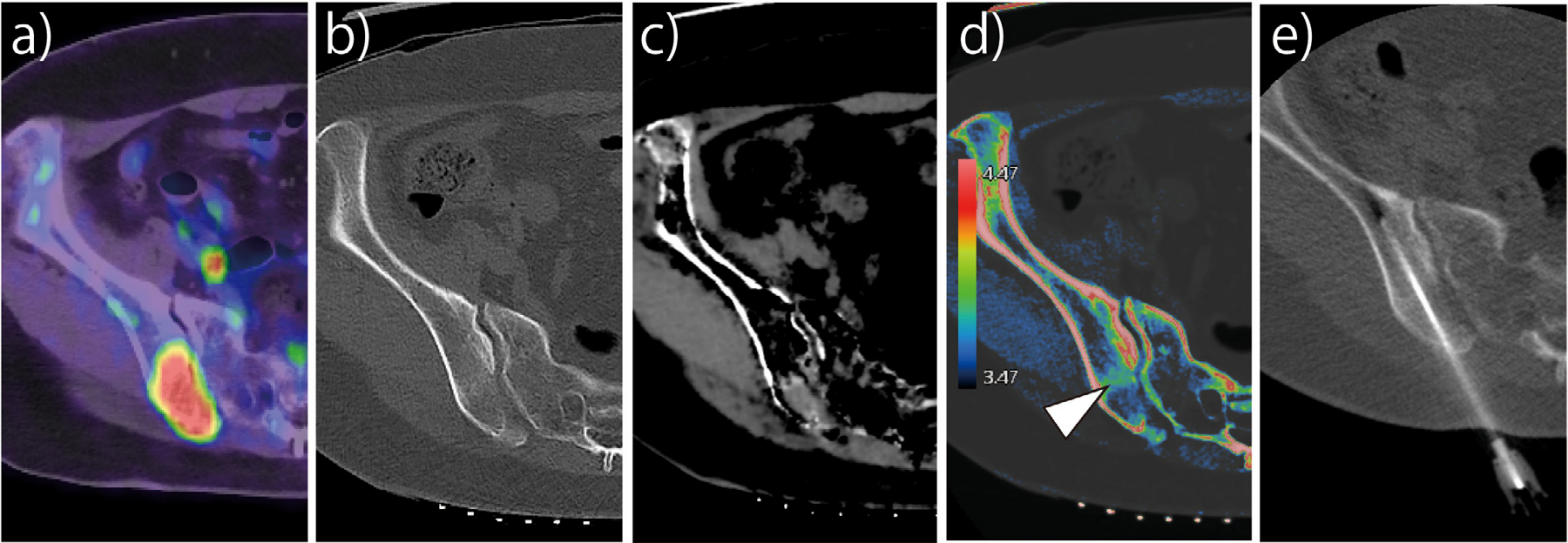
Images of the patient's right ilium. (a) An ^18^F-fluorodeoxyglucose positron emission tomography/computed tomography (FDG-PET/CT) image. The maximum standardized uptake value was 15.3. The patient's blood sugar at the time of imaging was 106 mg/dL. (b) A CT image to reveal bone condition. (c) Calcium suppression image (a virtual non-calcium technique). (d) Electron density map. *The high cellularity region showed high electron density (white arrowhead).* (e) A CT-guided bone biopsy image.

A 61-year-old woman with a history of breast cancer surgery was admitted to our hospital for a bone biopsy. Nine months prior to hospitalization, she was diagnosed with breast cancer using needle biopsy in her left breast. Imaging studies revealed bilateral breast cancer with left sentinel lymph node metastases. Five months prior to hospitalization, she underwent bilateral partial mastectomy and sentinel lymph node dissection. The breast cancer on her left side was an invasive ductal carcinoma. Four or more metastases of left axillary lymph nodes were identified to be Stage ⅢA (T2N2M0; expression of estrogen receptor: 95%; human epidermal growth factor receptor 2: negative; progesterone receptor: 25%; Ki-67: 20%). The breast cancer on her right side was determined to be a ductal carcinoma in situ. Three months prior to hospitalization, letrozole (2.5 mg/day) treatment was initiated as adjuvant therapy. Radiotherapy (total dose of 40 Gy) was performed on both sides of the breast. One month prior to hospitalization, the patient experienced left shoulder joint and chest pain, and sciatica. Ten days prior to hospitalization, ^18^F-fluorodeoxyglucose positron emission tomography/CT (FDG-PET/CT) revealed abnormal uptake in the systemic multiple bone, left axillary lymph node, and liver (Fig. 1**a**).

**Fig. 2:**
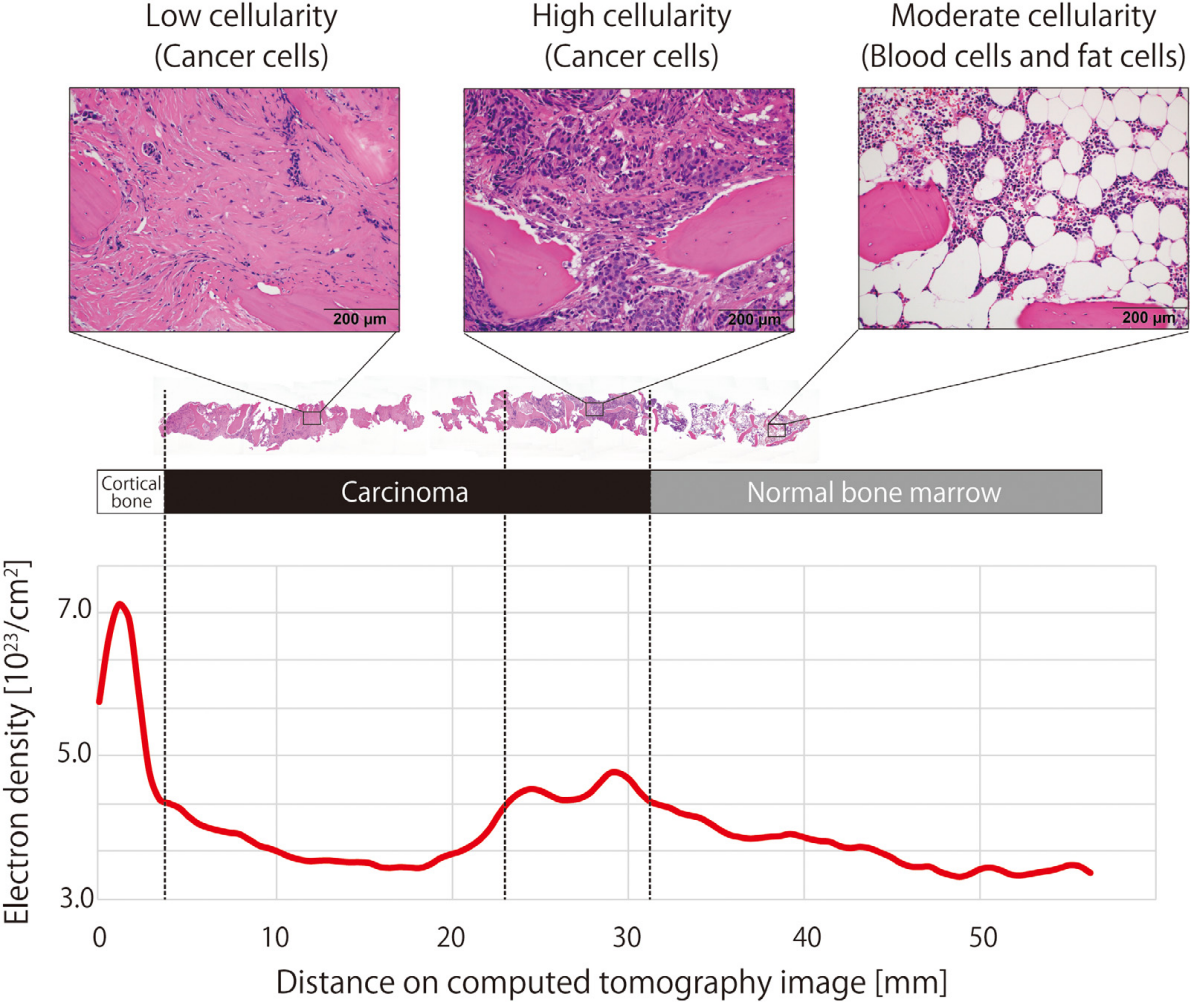
A pathological specimen and electron density spectrum A substantial level of cancerous foci growth in bone tissue was observed, which was consistent with metastatic breast cancer. The high cellularity area occurred between the normal bone marrow and a low cellularity area with fibrosis. Electron density is maximal in the cortical bone area (7.02 × 10^23^/cm^2^) and lower in the areas surrounding the high cellularity region (4.47 × 10^23^/cm^2^).

The patient's physician suspected multiple breast cancer metastases to the bone, malignant lymphoma, multiple myeloma, or other diseases. On the day of admission, a CT-guided bone biopsy of the right ilium was performed using a 10-cm, 13G Osteosite bone biopsy needle (Cook Medical, Bloomington, IN) (Fig. 1b–e). The biopsy procedure was performed via CT fluoroscopy, as previously reported [3], using an Aquilion ONE PRISM Edition CT, Canon Medical Systems Corporation, Tochigi, Japan (320-row detector; tube voltage: 135/80 kV; rotation speed: 0.75 s/rotation; collimation: 0.5 mm × 80; pitch factor: 0.637; tube current: AEC [SD: 6.0]; reconstruction, spectral body and spectral bone standard: determined by software). The total level of radiation to which the patient was exposed during the procedure was a 622.3 mGy × cm dose-length product. *The procedure time was 22 minutes*. We relied on an ED map created via DECT during the biopsy procedure. Both FDG-PET/CT and calcium suppression imaging revealed ^18^F-FDG uptake and a high signal value in the dorsal right iliac region, but the precise location of the high signal value was only revealed via ED mapping, approximately 3 cm from the bone surface. As a result of these analyses, the patient was diagnosed with breast cancer metastases to intertrabecular bone. The pathological specimen showed a low cellularity area with strong fibrosis that was approximately 2 cm from the bone surface, and high cellularity tumor cells approximately 3 cm from the bone surface. Normal bone marrow was observed at levels deeper than 3 cm from the bone surface (Fig. 2). A high ED of 4.47 × 10^23^/cm^2^ was observed only in the area with high cellularity (Fig. 2).

## Discussion

Bone metastases derived from breast cancer cells have been identified in 49% (range: 42-73%) of pooled and analyzed data from 10,521 patients [9], of which 22% are intertrabecular bone metastases [10]. Intertrabecular bone metastases, occasionally called CT-negative bone metastases [10,11], are difficult to diagnose from CT scans of bone condition (Fig. 1b). We were able to detect intertrabecular bone metastases before biopsy using ED map values. To the best of our knowledge, this is the first report that described a successful CT-guided biopsy using ED maps created via DECT.

In CT-guided biopsy, it is important to obtain a sufficient number of tumor cells for genetic testing or detection of hormone receptor expression [12]. FDG-PET/CT and calcium suppression images overestimate the dimensions of metastatic bone lesions (Fig. 1a, c). However, ED maps were the only imaging modality we used that accurately identified the extent of tumor cell spread (Fig. 1d). FDG-PET/CT data reflect glucose metabolism levels in tumor cells. Therefore, if tumor cell numbers are low and glucose metabolism is hyperactive, uptake levels likely increase, as was observed in this case. Use of calcium suppression imaging (a virtual calcium-removed technique) via DECT to evaluate acute fractures is well documented [13,14]. However, the technique is primarily used to assess bone marrow edema. In addition to imaging technologies, contrast media [3] and rapid on-site cytologic evaluation (ROSE) [15] have been used to confirm the presence of tumor cells during a biopsy procedure. However, contrast media cannot be used in patients allergic to the materials or in those with contrast medium-induced nephropathy [16], and ROSE requires knowledge of pathology and skill with regard to specimen preparation [15,17]. Currently, it is difficult to determine procedural success or failure during a CT-guided biopsy procedure. Therefore, a method that determines the presence of viable tumor cells during a biopsy procedure is of great value.

DECT provides ED maps due to the Compton effect of X-rays [5,18,19]. ED is determined by constituent atoms, bonds between molecules, and phase of matter [18,20,21]. In human bone, ED increases as bone mineral density increases, and ED decreases with the proportion of adipose tissue in the bone marrow increases [19,22,23]. A clinical study reported a method that can be used to determine the malignancy and cellularity of gliomas using ED maps [8]. Researchers were able to determine malignancy and cellularity since nucleic acids, prevalent molecular conjugate systems in cells of the human body, have a high ED due to short bond distances within the molecules [24]. Since healthy red bone marrow contains adipose tissue, it has a lower nucleic acid density than other parenchymatous organs or muscles [19,22,23]. In intertrabecular bone metastases, tumor cells proliferate to fill the intertrabecular space, eventually increasing the density of nucleic acids above that of normal bone marrow. Therefore, the ED maps obtained by DECT have the potential to accurately identify metastases with high cellularity in bone lesions.

## Financial/nonfinancial disclosures

None declared.

## Role of sponsors

None declared.
